# Supplementary material for: Distribution and demographics of mysids (Crustacea: Mysida) as prey for gray whales (Eschrichtius robustus) in northwest Washington state
Source: PeerJ. 2024 Jan 15;12:e16587. doi: 10.7717/peerj.16587 (PMC10795525; doi:10.7717/peerj.16587)
Supplement: Supplemental Information 1 — Total is the sum of all observations over both years. 2019 Per Sample and 2020 Per Sample show the average number of that taxonomic group collected per sample during each year. “Unk” is an abbreviation of “unknown” and is used to indicate that the organism was unable to be identified lower than the presented designation due to damaged identification features or lack of access to specialized expertise. [file peerj-12-16587-s001.docx]

| Taxon | |  | Total | Per Sample | | |  |
| --- | --- | --- | --- | --- | --- | --- | --- |
| Year | |  |  | 2019 | 2020 | |  |
| Annelida | | |  |  | |  |  |
|  |  | Polychaete | 9 | 0.07 | | 0.05 |  |
|  |  | Unk worm | 9 | 0.13 | |  |  |
| Arthropoda | | |  |  | |  |  |
|  | *Amphipods* | |  |  | |  |  |
|  |  | Caprellidae | 246 | 2.83 | | 0.54 |  |
|  |  | Grammaridae | 1 | 0.01 | |  |  |
|  |  | Unk Amphipod | 544 | 5.99 | | 1.45 |  |
|  | *Barnacles* | |  |  | |  |  |
|  |  | *Pollicepes polymerus* | 2 | 0.03 | |  |  |
|  |  | Unk barnacle nauplii | 36 | 0.50 | |  |  |
|  | *Crabs* | |  |  | |  |  |
|  |  | Cancridae | 3 | 0.03 | | 0.01 |  |
|  |  | Epialtidae | 6 | 0.33 | | 0.17 |  |
|  |  | Grapsidae | 37 | 0.25 | | 0.01 |  |
|  |  | Lithodidae | 19 | 0.10 | | 0.44 |  |
|  |  | Majidae | 41 | 0.17 | | 0.04 |  |
|  |  | Paguridae | 15 | 9.29 | | 10.47 |  |
|  |  | Pinnotheridae | 1486 | 7.31 | | 20.41 |  |
|  |  | Porcellanidae | 2118 | 0.08 | |  |  |
|  |  | Unk crab | 56 | 0.78 | |  |  |
|  |  | Xanthidae | 1 | 0.01 | |  |  |
|  | *Mysids* | |  |  | |  |  |
|  |  | *Columbiaemysis ignota* | 114 | 1.38 | | 0.19 |  |
|  |  | *Eucopia grimaldii* | 14 | 0.03 | |  |  |
|  |  | *Exacanthomysis davisi* | 2 | 0.19 | |  |  |
|  |  | *Hippacanthomysis platypoda* | 22 | 0.29 | | 0.01 |  |
|  |  | *Holmesimysis sculpta* | 11399 | 91.17 | | 61.99 |  |
|  |  | *Neomysis rayii* | 4348 | 60.10 | | 0.27 |  |
|  |  | *Telacanthomysis columbiae* | 92 | 1.28 | |  |  |
|  |  | Unk mysid | 192 | 2.58 | | 0.08 |  |
|  | *Shrimps* | |  |  | |  |  |
|  |  | Crangonidae | 7 | 0.10 | |  |  |
|  |  | Heptacarpus | 43 | 0.14 | | 0.42 |  |
|  |  | Hippolytidae | 20 | 0.28 | |  |  |
|  |  | Pandalidae | 79 | 0.94 | | 0.14 |  |
|  |  | Unk shrimp | 35 | 0.03 | | 0.42 |  |
|  | *Other* | |  |  | |  |  |
|  |  | Copepoda | 242 | 3.33 | | 0.03 |  |
|  |  | Euphausiacea | 28 | 0.39 | |  |  |
|  |  | Hippidae | 66 | 0.90 | | 0.01 |  |
|  |  | Isopoda | 225 | 2.58 | | 0.50 |  |
|  |  | Unk arthropod | 7 | 0.07 | | 0.03 |  |
|  |  | Unk Crustacean | 4 | 0.06 | |  |  |
| Cnidaria | | |  |  | |  |  |
|  |  | Unk Cnidarian | 2 | 0.03 | |  |  |
| Ctenophora | | |  |  | |  |  |
|  |  | *Pleurobrachia bachei* |  | 2.08 | | 1.28 |  |
| Mollusca | | |  |  | |  |  |
|  | *Snails* | |  |  | |  |  |
|  |  | *Calliostoma ligatum* | 2 | 0.03 | |  |  |
|  |  | *Fusitriton oregonensis* | 1 | 0.01 | |  |  |
|  |  | Littorinidae | 1 | 0.01 | |  |  |
|  |  | *Nucella lamellosa* | 2 | 0.03 | |  |  |
|  |  | *Olivella biplicata* | 2 | 0.03 | |  |  |
|  |  | *Tegula funebralis* | 5 | 0.07 | |  |  |
|  |  | Unk snail | 58 | 0.58 | | 0.21 |  |
|  | *Other* | |  |  | |  |  |
|  |  | Unk bivalve | 1 | 0.01 | |  |  |
| Teleosti | | |  |  | |  |  |
|  |  | Cottidae | 4 | 0.06 | |  |  |
|  |  | *Gobiesox maeandricus* | 4 | 0.06 | |  |  |
|  |  | Liparidae | 6 | 0.06 | | 0.03 |  |
|  |  | Unk flatfish | 1 |  | | 0.01 |  |
|  |  | Unk juvenile fish | 35 | 0.40 | | 0.08 |  |
|  |  | Unk larval fish | 9 | 0.13 | |  |  |
| Other | |  |  |  | |  |  |
|  |  | Unidentified | 43 | 0.60 | |  |  |
