# Supplementary material for: Distribution and demographics of mysids (Crustacea: Mysida) as prey for gray whales (Eschrichtius robustus) in northwest Washington state
Source: PeerJ. 2024 Jan 15;12:e16587. doi: 10.7717/peerj.16587 (PMC10795525; doi:10.7717/peerj.16587)
Supplement: Supplemental Information 2 — Unknown mysids were unable to be identified to species due to damaged or missing telsons. [file peerj-12-16587-s002.docx]

| Year | Month | *Holmesimysis sculpta* | *Neomysis rayii* | *Columbiaemysis ignota* | *Telacanthomysis columbiae* | *Hippacanthomysis platypoda* | *Eucopia grimaldii* | *Exacanthomysis davisi* | Unknown |
| --- | --- | --- | --- | --- | --- | --- | --- | --- | --- |
| 2019 | 6 | 0.4 |  |  |  |  |  |  |  |
| 2019 | 7 | 0.75 |  |  |  |  |  |  | 3.75 |
| 2019 | 8 | 89.28 | 14.11 | 0.06 |  |  |  |  | 3.39 |
| 2019 | 9 | 74.92 | 93.88 | 3.08 | 0.17 | 0.29 | 0.46 | 0.08 | 2.92 |
| 2019 | 10 | 174.77 | 35.15 | 0.08 | 6.08 | 1.08 |  |  | 2.00 |
| 2019 | 11 | 110.25 | 170.38 | 2.88 | 1.13 |  | 0.375 |  | 1.75 |
| 2020 | 6 | 47.00 | 3.00 | 0.5 |  |  |  |  |  |
| 2020 | 7 | 128.14 | 0.27 | 0.32 |  | 0.03 |  |  | 0.16 |
| 2020 | 8 |  | 0.24 | 0.10 |  |  |  |  |  |
| 2020 | 9 |  |  |  |  |  |  |  |  |
